# DNA barcoding Brooklyn (New York): A first assessment of biodiversity in Marine Park by citizen scientists

**DOI:** 10.1371/journal.pone.0199015

**Published:** 2018-07-18

**Authors:** Christine Marizzi, Antonia Florio, Melissa Lee, Mohammed Khalfan, Cornel Ghiban, Bruce Nash, Jenna Dorey, Sean McKenzie, Christine Mazza, Fabiana Cellini, Carlo Baria, Ron Bepat, Lena Cosentino, Alexander Dvorak, Amina Gacevic, Cristina Guzman-Moumtzis, Francesca Heller, Nicholas Alexander Holt, Jeffrey Horenstein, Vincent Joralemon, Manveer Kaur, Tanveer Kaur, Armani Khan, Jessica Kuppan, Scott Laverty, Camila Lock, Marianne Pena, Ilona Petrychyn, Indu Puthenkalam, Daval Ram, Arlene Ramos, Noelle Scoca, Rachel Sin, Izabel Gonzalez, Akansha Thakur, Husan Usmanov, Karen Han, Andy Wu, Tiger Zhu, David Andrew Micklos

**Affiliations:** 1 DNA Learning Center, Cold Spring Harbor Laboratory, Cold Spring Harbor, New York, United States of America; 2 Department of Herpetology, American Museum of Natural History, New York, New York, United States of America; 3 New York University, New York, New York, United States of America; 4 The New York Botanical Garden, Bronx, New York, United States of America; 5 The Rockefeller University, New York, New York, United States of America; 6 Genovesi Environmental Study Center, New York City Department of Education, Brooklyn, New York, United States of America; 7 CSI for International Studies, New York City Department of Education, Staten Island, New York, United States of America; 8 High School for Construction Trades, Engineering and Architecture, New York City Department of Education, Queens, New York, United States of America; 9 International High School at Union Square, New York City Department of Education New York, New York, United States of America; 10 High School for Health Professions and Human Services, New York City Department of Education, New York, New York, United States of America; 11 Frank McCourt High School, New York City Department of Education, New York, New York, United States of America; 12 Franklin D. Roosevelt High School, New York City Department of Education, Brooklyn, New York, United States of America; 13 Stuyvesant High School, New York City Department of Education, New York, New York, United States of America; 14 Forest Hills High School, New York City Department of Education, Queens, New York, United States of America; 15 Brooklyn International High School, New York City Department of Education, Brooklyn, New York, United States of America; Scientific Research Centre of the Slovenian Academy of Sciences and Art, SLOVENIA

## Abstract

DNA barcoding is both an important research and science education tool. The technique allows for quick and accurate species identification using only minimal amounts of tissue samples taken from any organism at any developmental phase. DNA barcoding has many practical applications including furthering the study of taxonomy and monitoring biodiversity. In addition to these uses, DNA barcoding is a powerful tool to empower, engage, and educate students in the scientific method while conducting productive and creative research. The study presented here provides the first assessment of Marine Park (Brooklyn, New York, USA) biodiversity using DNA barcoding. New York City citizen scientists (high school students and their teachers) were trained to identify species using DNA barcoding during a two–week long institute. By performing NCBI GenBank BLAST searches, students taxonomically identified 187 samples (1 fungus, 70 animals and 116 plants) and also published 12 novel DNA barcodes on GenBank. Students also identified 7 ant species and demonstrated the potential of DNA barcoding for identification of this especially diverse group when coupled with traditional taxonomy using morphology. Here we outline how DNA barcoding allows citizen scientists to make preliminary taxonomic identifications and contribute to modern biodiversity research.

## Introduction

DNA barcoding, or sequence-based specimen identification, was developed by Paul Hebert in 2003 to identify a broad range of taxa by sequencing a standardized short DNA fragment, the “DNA barcode” [1,2]. This technique has enabled the construction of the International Barcode of Life (www.iBOL.org) project, which has so far identified unique genetic barcodes for over 592,000 of the estimated 1 to 6 billion species on Earth [3,4]. For animals, the standard region to create a DNA barcode is the variable 5' half of mitochondrial gene cytochrome *c* oxidase 1 (*CO1*) [1,5]. A region of the chloroplast gene ribulose-1,5-bisphosphate carboxylase/oxygenase large subunit (*rbcL*) is used for barcoding plants [6], and fungi are barcoded using the nuclear internal transcribed spacer (ITS) region [7,8].

DNA barcoding has many practical applications including identification of fraud in consumer products, furthering the study of taxonomy, and monitoring and accounting for Earth’s biodiversity. In this time of major biodiversity loss, there is a necessity to identify and catalogue organisms to establish the baseline biodiversity. This helps to monitor biodiversity and potentially counteract the disappearance of species, and this can be rapidly accomplished through DNA barcoding [9,10]. An organism can often be sampled non-invasively, with minimal damage to any voucher sample. DNA barcoding can also be used to discover marketplace replacements and identify products taken from endangered species [11–18]. For example, DNA barcodes were previously used to find that five of 23 samples of caviar purchased in New York City (NYC) were mislabeled, including three from threatened sturgeon species [19]. Genetic marketplace monitoring has continued to be a promising tool for detecting fraudulent black caviar present in the NYC area [20].

**Fig 1:**
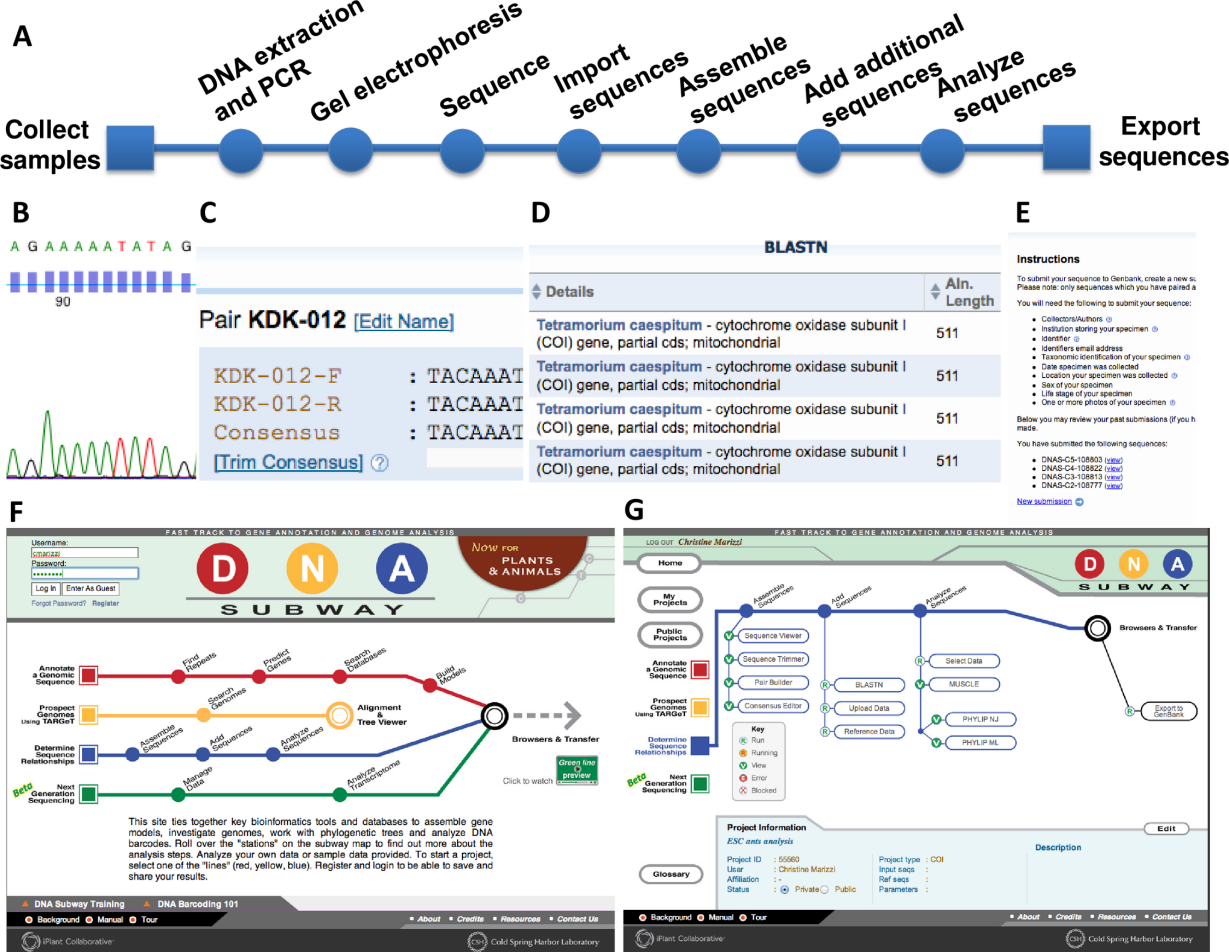
The DNA barcoding workflow. Biochemical protocols at the website *DNA Barcoding 101* (www.dnabarcoding101.org) and bioinformatics tools at *DNA Subway* (www.dnasubway.org) support all parts of the workflow. (A) Participants collected samples and extracted genomic DNA, generated DNA barcodes using PCR, verified the correct size by gel electrophoresis and sent amplicons for automated sequencing. Sequence data was uploaded to the internet-based DNA barcoding bioinformatics pipeline *DNA Subway*, and participants assembled contigs, compared them to additional sequences and analyzed sequence data for potential export to GenBank. (B) Trace file of a DNA sequence from a local ant sample. (C) DNA reads are paired and manually edited to create consensus sequence. (D) Top BLAST hits for an ant consensus sequence identifying it as *Tetramorium caespitum*. (E) *DNA Subway* integrated export function for novel DNA barcodes to GenBank. (F) User-friendly *DNA Subway* interface. (G) *DNA Subway’s* “Blue Line” for DNA barcoding and phylogenetics allows for sequence editing, performing a BLAST search, and phylogenetic tree building on an intuitive, open-source platform.

DNA barcoding is not just a powerful research tool, but the technique also allows students to complete authentic research projects in a short time period. The educational potential of DNA barcoding appeared when students from Trinity High School that documented mislabeling of seafood items purchased in NYC were featured in a media article [21]. Expanding on these efforts, the DNA Learning Center (DNALC) designed the *Urban Barcode Project* in 2011 to demonstrate that large numbers of high school students can work with their classroom teachers to complete independent research projects centered on DNA barcoding. The streamlined DNA barcoding curriculum (published online: www.dnabarcoding101.org) provides the infrastructure needed for teachers to replicate the DNA barcoding laboratory in schools or during workshops. The curriculum is largely based on the student-centered and discovery-based framework provided by the DNALC (see Fig 1).

**Fig 2:**
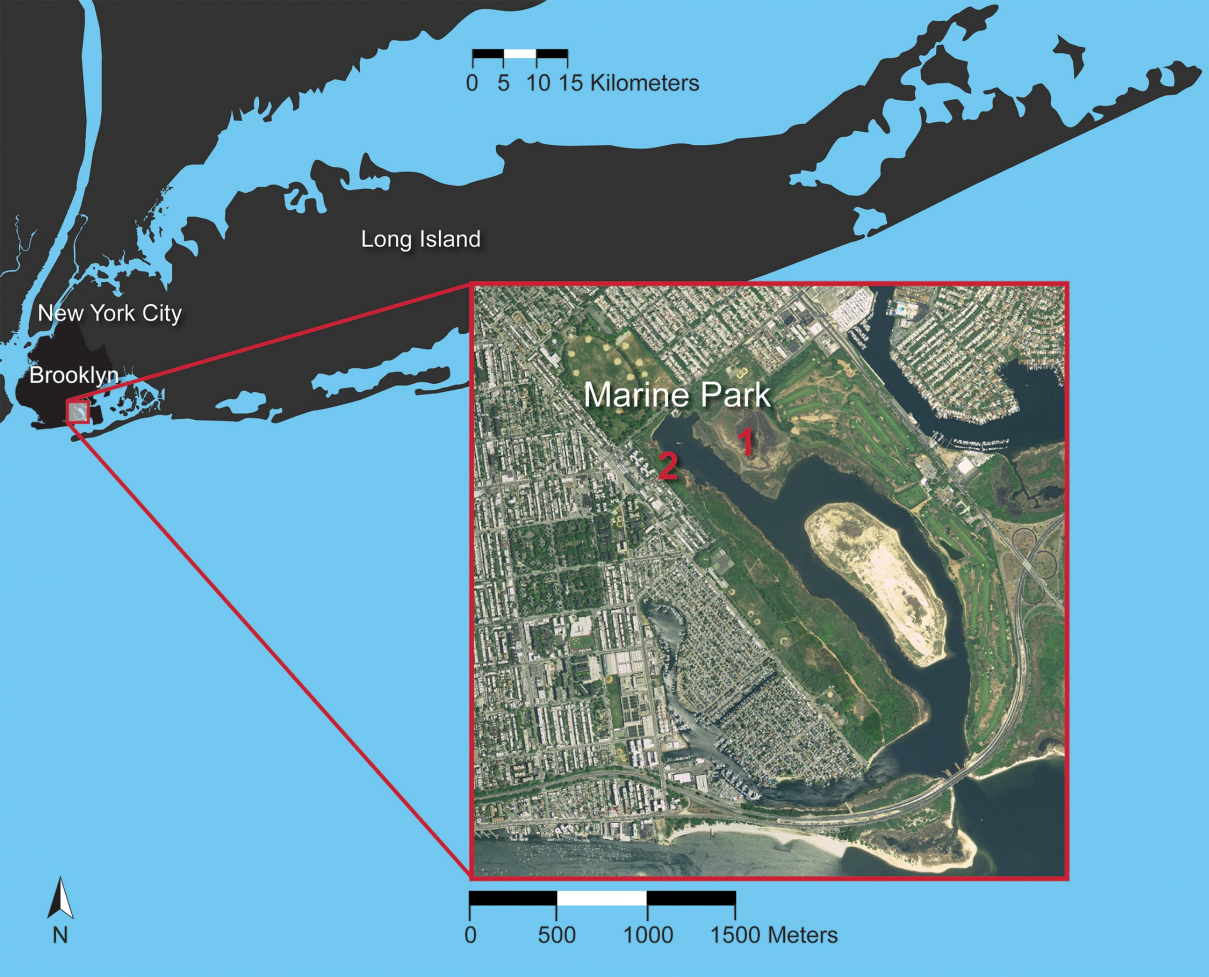
Sampling sites for this study. Samples were obtained at Marine Park (Brooklyn, New York, USA) in two localities: at a restored, more natural habitat (# 1 above) and an unrestored, disturbed habitat (#2 above). The figure was prepared based on data available from the U.S. Geological Survey Department of the Interior (USGS) public domain (USGS-viewer.nationalmap.gov) and edited by Chun-hua Yang, DNALC.

DNA barcoding programs offer the opportunity to bring students into natural areas, and students who participated in these workshops all spent time collecting samples as part of both a bioblitz and a targeted ant survey in Marine Park (Fig 2). Managed by the NYC Department of Parks and Recreation, Marine Park is the largest public park in Brooklyn and is protected as a Forever Wild Preserve. With more than 530 acres of grassland and salt marsh, and supplied with freshwater from Gerritsen Creek, Marine Park’s unique land (both natural and man-made) and water features provide an opportunity to study the effects of urbanization on wildlife in Brooklyn’s green areas [22].

While DNA barcoding introduces students to laboratory methods that will serve as a foundation to future research in academic labs, it also helps students develop an awareness and concern for biodiversity loss in NYC. Previous studies have suggested that this can be accomplished by inspiring a connection with nature [23,24] and by allowing students to develop a relationship with the species that surround them [25]. DNA barcoding in an educational setting achieves this because students are the lead investigators of their projects [26–29], and in doing so, become citizen scientists who are cataloging species biodiversity in NYC. This is an especially valuable lesson for urban students, like those in NYC, who are often disconnected from wildlife and the natural environment [30,31].

In partnership with the NYC Department of Education (NYCDOE) Genovesi Environmental Study Center (GESC), the DNALC’s barcoding curriculum was implemented during an immersive 10-day summer institute for high school teachers and their students in 2014 and 2015. Nine teachers from nine different NYC public schools, along with their team of students (3–4 students/teacher; 39 students total), were selected to participate in the institute held at GESC in Bergen Beach, Brooklyn. This institute provided teachers with a unique opportunity to attend professional development with their students. Together, they worked through the labs, data collection, and data analysis. This allowed teacher and student teams to start their research during the summer and continue it during the school year.

Teachers and their student teams began their DNA barcoding research project by exploring the local environment at Marine Park and the importance of the salt marsh ecosystem. The students completed research projects that fell into two categories: 1) a Marine Park bioblitz, where citizen scientist students collected all invertebrate and plant samples they found on designated sampling dates, and 2) the ants of Marine Park, where the focus was on collecting and cataloging ant species found in the park. For the ant project, the students worked with myrmecologist Dr. Sean McKenzie (Rockefeller University) to morphologically identify the samples, and then compared the morphological results to those found with DNA barcoding. Here we report all the results of these pilot projects where DNA barcoding was used as a foundation for introducing urban high school students to modern biodiversity research.

## Materials and methods

### Sample collection

A sampling permit was first obtained from the NYC Department of Parks and Recreation. Guided by Brooklyn Urban Park Rangers, as well as DNALC and GESC staff, students first visited the site and were taught to systematically document information as samples were collected. Sampling occurred July 14^th^, July 15^th^, July 25^th^, July 28^th^, July 29^th^ and August 4^th^ of 2014 and August 4^th^, August 5^th^ and August 10^th^ of 2015 (9 days total) for two hours each day. At the time of collection, all individual organisms were photographed, global positioning system (GPS) coordinates and locality information were recorded, and preliminary species identifications were made using field guides [32,33]. Students collected no more than five individuals per species and locality to prevent any detrimental impacts on the environment, species compositions, and population sizes. Ants were collected using Keebler® Sandies® Pecan Shortbread as bait, following School of Ants guidelines (http://schoolofants.org).

To generate a long-term record, sample metadata were entered into the DNALC Sample Database (https://sampledb.dnalc.org/barcoding), which functions as an online laboratory manual that can be accessed by the whole team to update and modify data (e.g., for data submissions to GenBank). Students were trained in preserving specimen vouchers for long-term storage. Vouchers were either frozen at -20°C directly (plants) or preserved in 100% ethanol (EtOH) [34] and subsequently frozen at -20°C (invertebrates). All samples were frozen within four hours of collection to minimize DNA degradation.

### DNA extraction, polymerase chain reaction (PCR), and sequencing

DNA extraction, PCR, and sequencing protocols are published under dnabarcoding101.org for teachers and professors to use worldwide. Here, we summarize these methods. For DNA extraction, a small portion of each sample (animal or plant) was placed in a 1.5 mL centrifuge tube, and ground using a plastic pestle in 300 μl lysis solution (6 M guanidine hydrochloride (Sigma-Aldrich, USA)). After incubating the sample for ten minutes at 65°C, samples were centrifuged for one minute at 6000 rcf and 150 μL of the supernatant was transferred to a fresh tube. Three μl silica resin solution (25 g of silicon dioxide (Sigma-Aldrich, USA, m.w. = 60.08) in 50 mL total volume of distilled water) was added to bind genomic DNA, followed by a five minutes incubation at 57°C. The samples were centrifuged again for one minute at 6000 rcf, the supernatant discarded and the silica pellet washed twice with 500 μL ice-cold wash buffer (20mM Tris-HCl pH 7.4, 50mM NaCl, 1mM ethylenediaminetetraacetic acid (EDTA), 50% EtOH). Finally the supernatant was discarded and DNA eluted in 100 μL distilled water by incubating samples for five minutes at 57°C.

The *COI*, *rbcL* and ITS barcode regions were amplified via PCR using illustra PuReTaq Ready-To-Go PCR Beads (GE Healthcare Life Sciences, USA). All PCR reactions were performed in a total volume of 25 μL, including 2.5 μl of template DNA, 1 μL of each primer (0.26 picomoles/μL), 2.5 units of Taq DNA polymerase, 10mM Tris-HCl (pH 9.0), 50mM KCl, 1.5mM MgCl_2_, 200μM of each dNTP, 13.8% sucrose, 0.0081% Cresol Red (Sigma-Aldrich, USA) and 22.5 μL of distilled water.

Invertebrate samples were amplified with DNA primers of the mitochondrial *COI*, LCO1490 (5’-TGTAAAACGACGGCCAGTGGTCAACAAATCATAAAGATATTGG-3’), and HCO2198 (5’-CAGGAAACAGCTATGACTAAACTTCAGGGTGACCAAAAAATCA-3’) [5]. The PCR conditions for the *COI* gene using LCO1490 and HCO2198 primers were as follows: initial denaturation at 94°C for one minute, 30 cycles of denaturation at 95°C for 30 seconds, annealing at 50°C for 30 seconds and extension at 72°C for 45 seconds. Plant samples were amplified with DNA *rbcL* gene primers rbcLaf (5' -TGTAAAACGACGGCCAGTATGTCACCACAAACAGAGACTAAAGC-3’) and rbcLarev (5’ CAGGAAACAGCTATGACGTAAAATCAAGTCCACCRCG-3’) [6,8]. The PCR conditions for the *rbcL* gene were as follows: initial denaturation at 94°C for one minute, 35 cycles of denaturation at 94°C for 15 seconds, annealing at 54°C for 15 seconds, and extension at 72°C for 30 seconds. Fungi samples were amplified with DNA primers ITS1F (5'-TGTAAAACGACGGCCAGTCCGTAGGTGAACCTGCGG-3') and ITS4 (5'-CAGGAAACAGCTATGACTCCTCCGCTTATTGATATGC-3') targeting the nuclear ITS region [7,35,36]. The PCR conditions were as follows for ITS1F and ITS4: initial denaturation at 94°C for one minute, followed by 35 cycles of denaturation at 94°C for one minute, annealing at 55°C for one minute and extension at 72°C for two minutes. Successful PCR products were confirmed by 2% agarose gel electrophoresis and stained with GelRed™ (Biotium, USA). To streamline sequencing the primers used in this experiment, we incorporated a universal M13 primer sequence at the 5’ end (M13F(-21): TGTAAAACGACGGCCAGT and M13R(-27): CAGGAAACAGCTATGAC) [37]. Sequencing was performed with an Applied Biosystems ABI 3730xl DNA Analyzer (ThermoFisher Scientific, USA) automatic sequencer at Genewiz (South Plainfield, NJ, USA).

### Data analysis

Sequences were analyzed using the “Blue Line” feature on *DNA Subway*, which supports phylogenetic and DNA barcode analyses [38,39]. A series of steps were used to ensure that only high quality data were used in subsequent analyses. First, DNA trace files were analyzed by Phred software [40,41], which calls a nucleotide (A, G, C, or T) for each peak. Each nucleotide is also assigned a “Phred” quality score that corresponds to a logarithmic error probability that the nucleotide call is wrong, or conversely, to the accuracy of the call (see http://www.dnabarcoding101.org/lab/bioinformatics.html). These quality scores for individual base calls are represented by blue bars on the *DNA Subway* sequence viewer. Nucleotides were only called if the Phred scores met or exceeded the quality cutoff (Phred score of 20, or greater than 99% accuracy, within a sliding window of 18, indicated with a blue horizontal line placed across the blue bars). Sequences with average Phred scores below 20 were flagged as “low quality”. Although this process is automated on *DNA Subway*, students were also taught to manually inspect each sequence to ensure that they agreed with the calls made by *DNA Subway*.

Next, sequences were trimmed in bulk by the default trimming function that removed “Ns” on the 5’ and 3’ prime ends (leaving in a sliding window of 12 nucleotides only one “N”) and automatically filtered out low quality sequences. Consensus sequences were built in *DNA Subway* using Merger—from EMBOSS (http://emboss.sourceforge.net/apps/release/6.6/emboss/apps/merger.html), and then all sequences were further manually edited to ensure primer removal.

After all of the above cleaning steps were completed, sequences were identified with a BLAST search on *DNA Subway* using default parameters, and then results were confirmed using a BLAST search directly on the GenBank website (https://blast.ncbi.nlm.nih.gov/Blast.cgi). The top BLAST result was determined using the highest percent identity score. In the event of a tie between two identity scores, the lowest e-value was used as a deciding-factor. If there were identical e-values and identity scores for multiple species, individuals were only identified to the class, family or genus level, to avoid any potential for incorrect taxonomic identifications.

Each sample was defined as “identified” if the highest percent identity score was ≥ 95%, and as a “potential new barcode” if the percent identity score was <95%. For sequences with ≥ 95%, after identification to genus and species-level on *DNA Subway*, higher-level plant taxonomic ranks (order, family and class) were determined from Tropicos (http://tropicos.org), and higher-level animal taxonomic ranks (order, class, and phylum) were determined from the Integrated Taxonomic Information System (http://www.itis.gov). Percentages of identified taxonomic groups were displayed using pie charts. For sequences of < 95% identity, each was carefully assessed to make sure that both reads were of high quality, with substantial overlap in the consensus sequence, and proper reading frames. If these characteristics were met, the sequences were submitted directly to GenBank as “novel barcodes” through the *DNA Subway* export function.

We also assessed the status of the identified genera and species as either native, invasive, or introduced to Marine Park. We did this using several guides by the New York State Department of Environmental Conservation (http://www.dec.ny.gov/animals/99141.html), the New York State Department of Agriculture and Markets [42], the Brooklyn Botanic Garden [43] and the USDA Forest Service [44]. An invasive species is a non-native species whose introduction does or is likely to cause economic or environmental harm or harm to human, animal, or plant health. Introduced or exotic species are also not native to the region, but differ from invasive species in that they do not cause significant damage to the ecosystem by outcompeting native species. (https://www.invasivespeciesinfo.gov/docs/council/isacdef.pdf). Additionally, morphological identification of selected ants collected in Marine Park was carried out by myrmecologist Sean McKenzie (Rockefeller University) using standard taxonomic keys [45,46]. Jeffry Petracca (Long Island Aquarium) morphologically identified any samples published as novel DNA barcodes.

## Results

### Assessment of species collected in Marine Park

**Fig 3:**
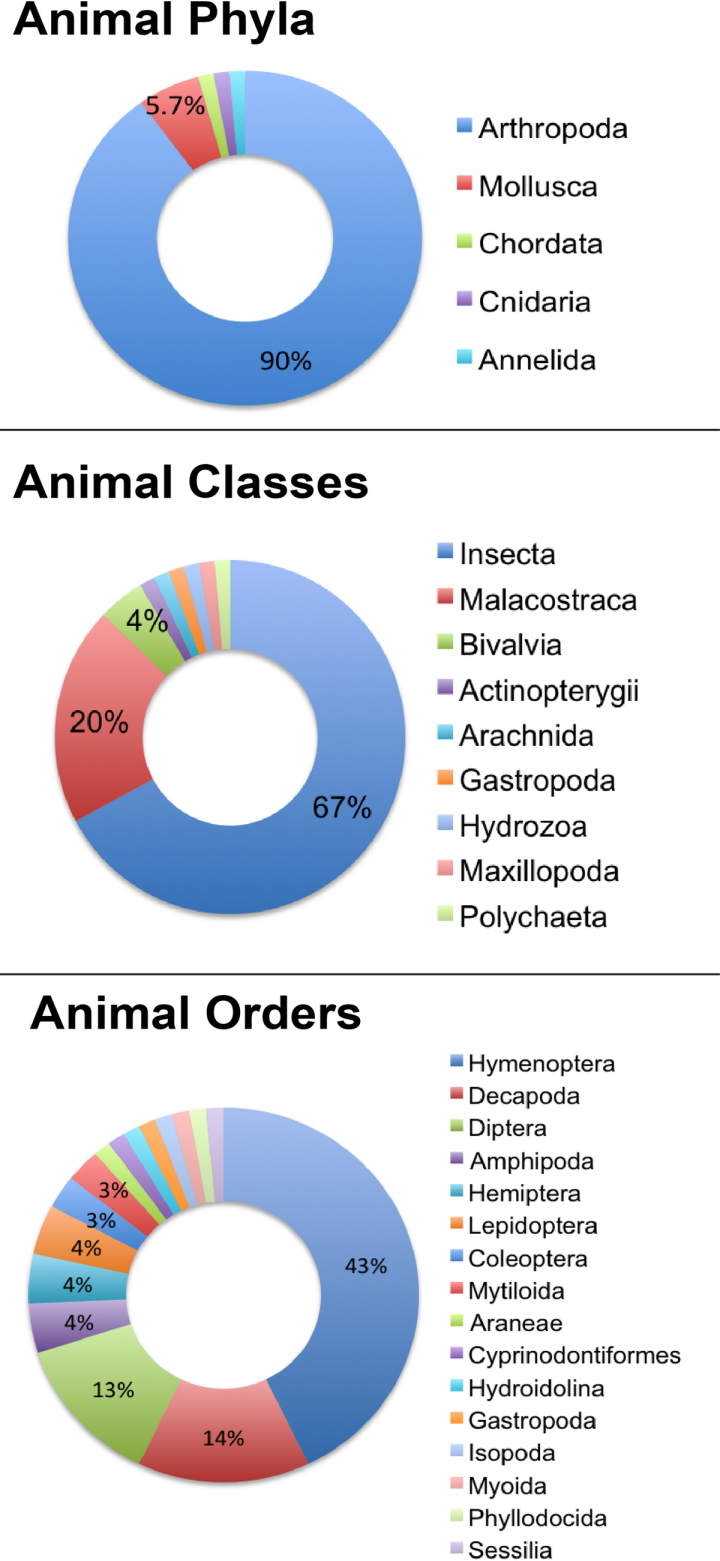
Overview of invertebrates identified by this project, organized by taxonomic level. The vast majority of the animal samples belonged to the phylum Arthropoda (n = 63; 90% of collected animals). Of those 63 samples, most were from the Insecta class (n = 47; 67.1% of collected animals) in the Hymenoptera (n = 30; 42.8% of collected animals) and Diptera (n = 9; 12.8% of collected animals) orders. Taxonomic groups in legends are organized from largest to smallest.

Over the two summers included in this study (2014 and 2015), student teams collected and identified 187 samples (1 fungus, 70 animals, and 116 plants; a full list of the samples, along with identification and sequence information can be found in S1 Table). Overall, we identified 87 genera and 98 unique haplotypes from the 187 samples collected in Marine Park (S2 Table). The sole fungus sample was identified as *Simocybe serrulata*, and an overview of the animal and plant samples are provided below. For the animal samples (see Fig 3), only one chordate was sequenced (sample KQB-011)–the fish *Fundulus heteroclitus* (mummichog, a small killifish species), provided by a park ranger–because collection was restricted to invertebrate samples. Ninety percent of the remaining samples were invertebrates belonging to the phylum Arthropoda (n = 63; 90% of collected animals). Of those 63 samples, most were from the Insecta class (n = 47; 67.1% of collected animals) in the Hymenoptera (n = 30; 42.8% of collected animals) and Diptera (n = 9; 12.8% of collected animals) orders (Fig 3). The second most abundant samples in the Arthropoda phylum were from the Malacostraca class (n = 14; 19.7% of all collected animals), of which ten (14.1% of total animals) were in the Decapoda order, three (4.2% of total animals) were Amphipods, and one (1.4% of total animals) was an Isopod. The remaining 8.6% of the total animals were from the phyla Mollusca, Cnidaria and Annelida.

**Fig 4:**
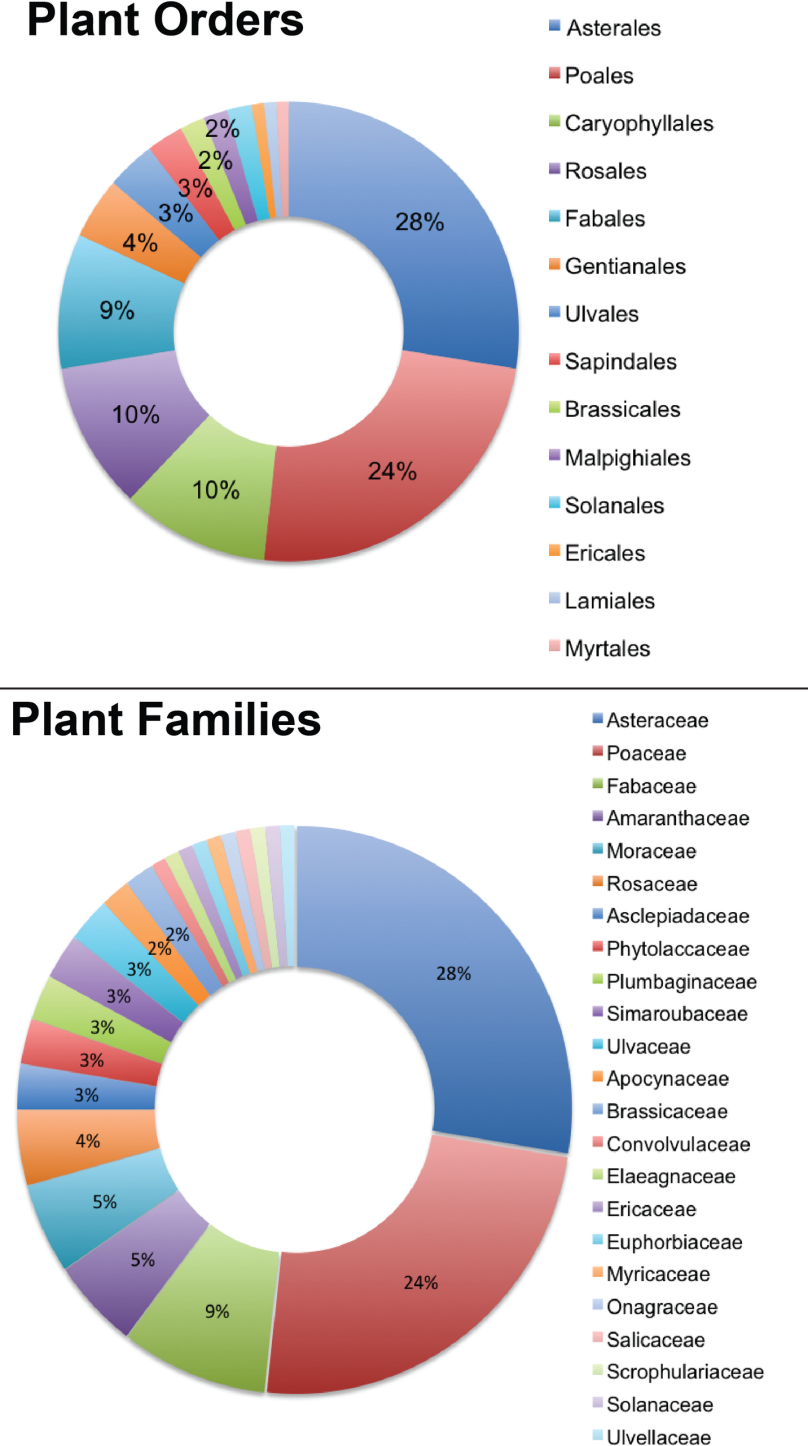
Overview of plants identified by this project, organized by taxonomic level. Of the 116 collected and identified plants the majority were in the Asterales (n = 32; 27.6% of total plants) and Poales (n = 28; 24.1% of total plants) orders, with the highest number of species within each order falling in the Asteraceae (n = 32; 27.6% of total plants) and Poaceae families (n = 28; 24.1%). Taxonomic groups in legends are organized from largest to smallest.

Of the 116 collected and identified plants, the majority were in the Asterales (n = 32; 27.6% of total plants) and Poales (n = 28; 24.1% of total plants) orders, with the highest number of species within each order falling in the Asteraceae (n = 32; 27.6% of total plants) and Poaceae families (n = 28; 24.1% of total plants; see Fig 4). We also found 24 plants in the Rosales and Carophyllales orders (each having n = 12; 10.3% of total plants).

The majority of the sequences analyzed (168/187) received high similarity scores (> 95%) on GenBank. From the remaining 19 samples with low similarity scores (<95%), 12 new barcodes were published as GenBank entries with participants as authors (see Tables 1 and S1 for full sample information), and each of these submissions were verified by a taxonomist. In most instances, we were conservative in our identifications and only identified samples to higher-order ranks (e.g. families or classes). Genus and species-level identification was only made when, in addition to no ties between identify and e-value scores on GenBank, an expert taxonomist confirmed that identity. The seven remaining samples with low similarity scores were not published as novel barcodes because the sequence quality was low and these samples were excluded from these analyses.

**Table 1:** Overview of the twelve novel DNA barcodes generated by the participants of the Urban Barcoding Project Institute at GESC in the summers of 2014 and 2015.

Sample name	Closest matching taxon name	Taxonomic level	Gene	GenBank accession code
GQB-003	Ephydridae	Family	*COI*	KU682771
GQB-004	Carabidae	Family	*COI*	KU682774
GQB-007	*Gnaphosa* sp.	Genus	*COI*	KU682773
GQB-064	Bombyliidae	Family	*COI*	KU682772
GQB-070	Leptothecata	Order	*COI*	KU682775
KDK-019	*Spartina* sp.	Genus	*rbcL*	KT956910
KDK-021	Ulvellaceae	Family	*rbcL*	KT959344
KDW-016	*Mya arenaria*	Species	*COI*	KT960977
KHN-010	*Monomorium* sp.	Genus	*COI*	KX711882
KHN-011	*Monomorium* sp.	Genus	*COI*	KX711883
KHN-012	*Nylanderia* sp.	Genus	*COI*	KX711884
KHN-013	*Monomorium* sp.	Genus	*COI*	KX711885

We also assessed whether the species we collected in Marine Park were native, introduced, or invasive based on our preliminary taxonomic identifications. From our collected animals, we found 19 native, five introduced, and one invasive species (the ailanthus webworm *Atteva aurea–*see S2 Table); the rest could not be classified based on the guides we used or the taxonomic level (e.g. some only identified to genus-level). Examples of native animal species to the U.S. Northeast region include the ground spider *Gnaphosa* sp., and the soft-shell clam *Mya arenaria*. Introduced animal species included the coccinellid beetle *Harmonia axyridis* and the amphipod *Grandidierella japonica*. From our collected plants, we found 20 native, 14 introduced, and four invasive species; the remainder could not be classified for the same reasons given above for the animal samples. Examples of abundant invasive plant species include the China-sumac *Ailanthus altissima*, the invasive genotype of the common reed *Phragmites australis* and the Autumn olive *Elaeagnus umbellata*. All other species that could not be classified into these categories are marked as unknown in S2 Table.

### The utility of barcoding genes

The ability of the traditional barcoding genes (*COI*, *rbcL*, and ITS) to identify samples varied across taxonomic groups when we were making our preliminary taxonomic assessments using GenBank. The one fungus sample (GQB-045) was identified to the species-level with ITS. In arthropods, 43/63 (68.2%) of samples could be identified to the species-level (see S1 Table). All four mollusk haplotypes, the one annelid, and the one chordate haplotype were sufficient to identify those samples to the species-level. The one cnidarian haplotype could only identify that sample to the genus level. Across all 70 animal samples, 49 samples (or 70%) were identified to the species level using preliminary taxonomic identifications based on GenBank searches.

Plant identification with *rbcL* was generally less successful than animal identification with *COI*. The largest numbers of samples were found in the Asterales and Poales orders. In Asterales, 14/32 of samples (43.8%) could be identified to the species-level, while in Poales 11/28 (39.3%) of samples could be identified to the species-level (see S1 Table). In all instances, sequence lengths were standard for DNA barcoding (>500 bp) with few nucleotide ambiguities. Across all 116 plant samples, 49 samples (or 42.2%) were identified to the species level using preliminary taxonomic identifications–see S1 Table.

### Barcoding ants in Marine Park

We collected 29 ant samples from seven ant species (all in the Formicidae family), and four of these were submitted as novel barcodes from the two ant genera *Nylanderia* and *Monomorium* (Table 1). We were interested in whether the DNA barcodes yielded identifications that were congruent with morphological identifications, and examined this using a subset of 20 samples (Table 2). The DNA barcode and morphological identifications were congruent in each case, but in some cases either barcoding or traditional taxonomy returned species-level identifications when the other method only identified the sample to the genus-level.

**Table 2:** List of ant samples, and their corresponding morphological and DNA barcoding identifications.

Sample name	Morphological identification	DNA barcoding identification
KFJ-019	*Tetramorium caespitum*	*Tetramorium caespitum*
KFJ-020	*Nylanderia* cf. *flavipes*	*Nylanderia* sp.
KFJ-021	*Tapinoma sessile*	*Tapinoma sessile*
KFQ-015	*Tetramorium caespitum*	*Tetramorium caespitum*
KFQ-017	*Monomorium* cf. *viride*	*Monomorium* sp.
KFQ-018	*Monomorium* cf. *viride*	*Monomorium* sp.
KFQ-019	*Tapinoma sessile*	*Tapinoma sessile*
KHN-003	*Tetramorium caespitum*	*Tetramorium caespitum*
KHN-004_1	*Tapinoma sessile*	*Tapinoma sessile*
KHN-004_2	*Nylanderia* cf. *parvula*	*Nylanderia* cf. *parvula*
KHN-006_1	*Nylanderia* cf. *parvula*	*Nylanderia* cf. *parvula*
KHN-006_2	*Tetramorium caespitum*	*Tetramorium caespitum*
KHN-007	*Tetramorium caespitum*	*Tetramorium caespitum*
KHN-008	*Tetramorium caespitum*	*Tetramorium* sp.
KHN-009	*Aphaenogaster* sp.	*Aphaenogaster rudis*
KHN-010	*Monomorium* cf. *viride*	*Monomorium* sp.
KHN-011	*Monomorium* cf. *viride*	*Monomorium* sp.
KHN-012	*Nylanderia* cf. *flavipes*	*Nylanderia* sp.
KHN-013	*Monomorium* cf. *viride*	*Monomorium* sp.
KHN-014	*Monomorium* cf. *viride*	*Monomorium* sp.

Each sample was identified both by performing a BLAST search on GenBank and then verified by a taxonomist. KHN samples: Barcodes generated by students from Forest Hills High School, Queens. KDK samples: Barcodes generated by students from High School for International Studies, Staten Island. GQB samples: Barcodes generated by students from Frank McCourt High School, Manhattan. KDW sample: Barcode generated by Christine Marizzi, DNALC.

In all cases, amateur high school taxonomists were able to use DNA barcoding to identify the same genus as the morphologist. In nine instances, DNA barcodes were unable to definitively identify the ant to the species-level.

## Discussion and outlook

Mirroring prior DNA barcoding student projects (unpublished data, student project descriptions and talks can be found at dnabarcoding101.org), the majority of novel barcodes never before published on GenBank were from invertebrate samples (10/12 novel barcodes, or 83%, in our study; Table 1). The most abundant invertebrates collected were insects, which is not surprising since insects have been predicted and found to be among the most abundant and speciose animal groups [46]. We will continue exploring this result, perhaps with curated campaigns looking for specific invertebrates, such as nematodes, which are also known to be very speciose and abundant [47,48]. In our study, the most abundant plants were in the Asteraceae and Poaceae families. Similar to what we found, Asteraceae is the most species rich and largest family of flowering plants [49], with Fabaceae the second most speciose family [50] and Poaceae the third most speciose family in North America (hhtp://bonap.net/tdc). Our finding an abundance of Poaceae species in Marine Park is not surprising since grasses are abundant in NYC parks.

An additional classification we made was to determine whether the species we identified were native, introduced, or if their status was unknown. While this is important information to provide a snapshot of the type of species living in an ecosystem, it is also a way to expand upon a traditional barcoding project for more advanced students. Students at the institute were encouraged to specifically look for introduced or invasive species, and several student teams gave presentations focused on these organisms. One example of a presentation done by a team was about the invasive variety of *Phragmites australis*, or common reed, which was found in high abundance in Marine Park. *Phagmites* species do particularly well in warmer wetland habitats, and some studies have suggested that its abundance may increase due to warmer temperatures that result from global climate change [http://www.nyis.info/index.php?action=invasive_detail&id=42]. Students can use DNA barcoding to help monitor and track the abundance of this invasive species over several years to see if this pattern holds true in Marine Park.

While using one universal marker for species-level identification has advantages because it is rapid, simple, and automatable [51], there are limitations. Previous reports showed that molecular analysis using conserved universal primers in the Folmer *COI* region have the potential to recover putative numt (nuclear mitochondrial pseudogene) sequences in addition to orthologous mtDNA, leading to DNA barcoding ambiguity [8,52,53]. We carefully screened for typical indicators of potential poor-quality data, such as multiple bands on a gel, raw sequence ambiguities, background noise and double peaks. Before publishing novel DNA barcodes to GenBank, sequences were translated to verify the absence of indels and stop codons using *DNA Subway* default settings. We found that *COI* was able to identify 70% of samples across all animal groups to the species-level, and the remainder to the genus-level with the exception of one sample. *COI* performed better than *rbcL*, and only 42.2% of plant samples could be identified to the species-level. The absence of a single plant barcode marker with both high amplification success rates and discrimination power poses a difficulty for rapid species identification [6,54]. When a sample cannot be identified to the species-level using a single barcoding gene, we recommend the use of morphology or additional markers, such as 28S rDNA, to confirm and pinpoint species identities [55,56]. Additionally, because *rbcL* does not often allow for species level identification in plants, many plant taxonomists now advocate also using *maturase-K* (*matk*) and nuclear ribosomal internal transcribed spacer (ITS) for barcoding seed plants [54,57,58]. While integrating the use of two loci will increase the cost of outreach programs, this approach would contribute toward the identification of samples to the species-level.

Another note of caution is that the NCBI database results must be carefully examined because some of the published sequences on GenBank are misidentified. For example, a study found that many of the species on GenBank are misidentified as metazoans, when they are in fact bacteria, because the use of universal primers can anneal to bacterial species in error [59]. For fungi, it has been reported that 27% of fungal ITS sequences on GenBank were inadequately identified and 20% were incorrectly labeled [60]. To ensure that any sequences we submitted were correct, we only submitted novel barcodes with two high quality sequence-reads to GenBank and had the identities confirmed by a taxonomist. We were also very conservative when it came to identifying any lower-level classifications, and instead often used higher-level taxonomic ranks (e.g., families or orders). This careful consideration before submission to GenBank is especially important for student-generated data because students may make errors during the wet laboratory and subsequent bioinformatics searches. This is also why establishing and maintaining accurate libraries that will help identify specimen is not only important for research purposes but also for science education.

We found that small-bodied animals that were most often collected by students, and that also yielded novel barcodes, were ants. This is not surprising, since ants are collected easily using cookies as bait (http://schoolofants.org), and students are often not morally opposed to euthanizing an ant species for DNA extraction (as opposed to larger invertebrates, like crabs). Students also often sampled close to noon, a time when the majority of ant species were foraging. The ants we found in highest abundance belonged to the *Monomorium*, *Tetramorium*, and *Nylanderia* genera. While the ant genus *Monomorium* has been described worldwide, about 26 of 400 species are found in North America (www.antwiki.org). *Tetramorium caespitum* is also known as the pavement ant and is an introduced pest in North America that is often found in high abundance, and was also found as very abundant in another urban habitat, NYC street medians [61]. The *Nylanderia* genus includes 110 species and has a nearly cosmopolitan distribution, with species inhabiting a wide array of habitats in almost all geographic regions, except Europe (www.antwiki.org) [62,63]. Both key introduced species *Tetramorium caespitum* and *Nylanderia flavipes* have been known to be present in NYC. Interestingly, the majority (5/7) of our collected ant species (*Tapinoma sessile*, *Nylanderia* cf. *flavipes*, *Aphaenogaster rudis*, *Lasius neoniger* and *Tetramorium* spp.) have been previously reported as being present in NYC parks; while two others (*Monomorium* cf. *viridae and Nylanderia* cf. *parvula* have not been found [61,64].

We were especially interested in comparing the DNA barcode ant species results with the identifications made by an expert ant taxonomist, and the results are shown in Table 2. In all cases, the *COI* gene is sufficient for identifying ant genera, and these identifications were congruent to the identifications made by the morphologist. Interestingly, no *Monomorium* samples could be identified to the species-level using DNA barcoding, and this genus was also difficult for morphological identification. For example, we were uncertain if we had collected *Monomorium viride* or another *Monomorium* species. Overall, this case study showed the utility of DNA barcodes for accurate identifications by amateur scientists at least to the genus-level.

The 39 students who participated in the summer institute were required to create presentations to report their findings to their peers and their instructors. Because science practices such as oral and poster presentation skills are a defining element of course-based research experiences (CUREs) [65] and are encouraged at all levels of STEM (science, technology, engineering, and math) education, we recommend incorporating this in all DNA barcoding programs.

## Conclusions

DNA barcoding continues to demonstrate its potential as a powerful tool for students to act as citizen scientists to make a real contribution to ecosystem assessment. Our findings support that when given the tools [66], high school students generated high-quality data and identified challenging taxonomic samples such as ants with the same accuracy as experts. In terms of species identification using GenBank alone, we showed that while *COI* was able to identify the 70% of animal samples to the species-level, only 42.2% of plant samples could be identified to the species-level. We recommend building additional plant DNA barcoding markers such as *matK* or ITS into the design of future biodiversity sampling programs to help pinpoint plant species identifications. We also caution about using GenBank alone to make definite species confirmations, and instead encourage the use of traditional taxonomy combined with DNA barcoding when submitting novel sequences to Genbank. Most collected plants were common flowering plants in the Asteraceae and Poaceae families (51.7%), and most animals were insects from the phylum Arthropoda (90%). Additionally, students identified two ant species that were previously unreported in a NYC public park and published 12 novel barcodes on GenBank, underlining that the established DNA barcoding workflow can be used to teach molecular techniques and bioinformatics in a contextual, situated learning design that is relevant personally and to the scientific community.

## Supporting information

S1 Table Complete list of 187 samples included in this study, along with sample names, taxonomic identification, sequence information, and species status. (XLSX)

S2 Table Complete list of taxonomic identifications made from the 187 samples included in this study. In some instances, we could not identify the samples to the genus-level.
(XLSX)
